# Nutritional status and correlation with academic performance among primary school children, northwest Ethiopia

**DOI:** 10.1186/s13104-018-3909-1

**Published:** 2018-11-09

**Authors:** Biachew Asmare, Mekuanint Taddele, Sileshi Berihun, Fasil Wagnew

**Affiliations:** College of Health Sciences, Debre Markos University, PO. Box: +251-269, Debre Markos, Ethiopia

**Keywords:** Academic performance, Nutritional status, School age children

## Abstract

**Objective:**

This study aimed to determine the association between nutritional status and academic performance among primary school children in Debre Markos Town, northwest Ethiopia, 2017.

**Results:**

The prevalence of stunting, underweight and wasting were 27.5% (95% CI 23.2–31.9%), 20.4% (95% CI 16.5–24.3%) and 8.7% (95% CI 6.2–11.5%), correspondingly. The low level of educational performance was significantly higher (p < 0.05) among the stunted, underweight and wasted children than that of the normal children. In multivariable logistic regression, age of the child (Adjusted Odds Ratio (AOR) = 0.177, 95% CI 0.07, 0.4), monthly income less < 1000.00 birr (AOR = 0.05, 95% Cl 0.02, 0.15), stunted children (AOR = 0.21, 95% CI 0.10, 0.43) and under-weight (AOR = 0.63, 95% CI 0.26, 0.84) were associated with academic performance. This study revealed that indicators of undernutrition were prevalent among school-age children. Thus, collaboration between the health and education sectors is required to alleviate the problem.

**Electronic supplementary material:**

The online version of this article (10.1186/s13104-018-3909-1) contains supplementary material, which is available to authorized users.

## Introduction

Quality education plays a pivotal role in the economic, social and political development. Currently, getting children into schools is not enough; government ensure that children attain the basic knowledge and skills needed for personal well-being [1]. Primary school is an important stage in the development of consciousness and personality of the child [1, 2].

Nutrition is also a vital component of human health, life, and brain development through the entire lifespan [3]. Balanced nutritious is crucial for endurance, physical growth, cognitive development and productivity [4]. As well, malnutrition is considered a pressing problem that affects the ability of children to learn and causes them to perform at a lower level in school [5–7]. Undernutrition is a major public health challenging affecting academic school achievement [8]. Ethiopia is one of the sub-Saharan African countries basically affected by child malnutrition. Previous studies conducted in different areas have shown that under nutrition is common among school-age children; it was reported in the form of stunting range from 11 to 48.7% and underweight from 7.2 to 59.7% [9]. A study conducted in eastern Ethiopia reported that the prevalence of stunting was 8.9%, of which, 2% had severe stunted among school-aged children [10]. Though evidence about the prevalence of malnutrition is well studied in Ethiopia, there is insufficient evidence regarding nutritional status allied with academic performance among school-age children [9]. The association between nutritional status and educational achievement among school-age children in developing countries have not been recognized well [9, 11]. Stunting is referred as the best indicator for a chronic type of under nutrition [9]. Children who are stunted have low ability to learn at school and poor scholastic achievement [12]. Furthermore, poor feeding practices are associated with stunted and impaired brain development [6, 13].

On this background, there is a necessity to overlook the relationship between nutritional status and educational performance among school-age children in the Debre Markos town. This study was aimed to determine nutritional status and correlation with academic performance among first cycle governmental primary school in Debre Markos Town, northwest Ethiopia.

## Main text

### Methods

#### Study area, setting and period

The study was conducted at Debre Markos town primary school. In the town, there were a total of 7473 population. Of them, 3831 were females studying in the school. Debre Markos is a city of East Gojjam Zone which is located 299 km away from Addis Ababa in the North. It had 15 governmental and 8 private primary schools. The study was conducted between January15 to March 17/2018.

*Study design and population:* An institutional-based, cross-sectional study was employed at primary school in Debre Markos town.

*Sample size and sampling techniques:* The sample size was determined using double population proportion by considering the following statistical assumptions: prevalence of stunting among school children (p_1_) is 48% and p_2_ is 29% and level of significance (α) = 5%, at 95% level of confidence, power of the study 90% and design effect 1.5. Finally, the overall sample size was found 442.

*Sampling procedure:* Participants were carefully chosen using a multi- stage sampling technique. Out of 15 primary schools, 4 schools were selected randomly by lottery method at stage one. Students were allocated proportionally at stage two. Then participants from selected schools were selected by systematic random sampling method using students’ name list by calculating ‘k’ value for each class.

*Data collection methods:* Data were collected using a pre-tested structured questionnaire and translated into the local language (Amharic version) by trained and experienced data collectors. Respondents were parents/caregivers of the children identified in the study schools. After students were systematically selected from the schools, their household address was traced in the students’ parent database. Then data collectors went to the children’s house to interview parents/caretakers. Training on the standard procedures and technique how to collect data were given for the data collectors and supervisors for 2 consecutive days. The contents on questionnaires were briefly described to reduce interviewer bias.

*Data processing and analysis:* Data were entered into Epi-Data version 3.1 and then exported to SPSS version 20 for further analysis. Emergency Nutrition Assessment (ENA) for SMART software was used to calculate the Z-score of weight-for-age, height-for-age and weight -for-height of the children. Variables which were significant at p-value < 0.2 in the bivariable analyses were candidate for entering into the multivariable logistic regression model to identify the independent predictors for academic performance. Before inclusion of factors, we checked multicollinearity using variance inflation factor (VIF) < 10.

#### Definition of academic performance

The overall subjects the students were given in the academic year 2017/18 were considered to examine the academic achievements of the students. The annual average score was computed by taking the result of two consecutive semesters of the year. To verify the relationship between nutritional status and academic performance, average marks of the overall subjects the students received were either poor academic achievement or good academic achievement, in accordance with an average mark of 50%. This cut off average point was decided by considering the pass mark set by Ethiopian ministry of education.

### Results

#### Socio-demographic characteristics

A total of 436 children were included in the study with a response rate of 98.6%. Of them, 245 (56.2%) were males. The mean age of the study participants was 8.57 (± 1.12) ranging from 7 to 10 years. Majority of the study participants 398 (90.8%) were located in urban, 389 (89.2%) orthodox and 153 (35.1%) from grade one. Educational status of parents of the study participants showed that 81 (19.5%) mothers and 67 (17.7%) fathers had no formal education (Table 1).

**Table 1:** Socio-demographic characteristics of study participants in Debre Markos town, Northwest, Ethiopia, 2017 (n = 436)

Variable	Category	Frequency (N: 436)	Percent (%)
Residence	Urban	396	90.8
	Rural	40	9.2
Age of a child (years)	7	87	19.9
	8	122	28
	9	109	25
	10	118	27.1
Sex	Male	245	56.2
	Female	191	43.8
Religion	Orthodox	389	89.2
	Muslim	19	4. 4
	Protestant	25	5.8
	Others	3	0.6
With whom a child lives	Mother	37	8.5
	Father	14	3.2
	Mother and father	377	86.5
	Grand families	5	1.14
	Others	3	0.66
Marital status of Parents/caregivers	Single	17	3.9
	Married	367	84.2
	Divorced	37	8.5
	Separated	11	2.5
	Others	4	0.9
Educational status of mothers	No formal education	81	19.5
	Primary	162	39.2
	Secondary	133	32.2
	Diploma	27	6.5
	Degree and above	11	2.6
Fathers’ Educational status	No formal education	67	17.7
	Primary	94	24.9
	Secondary	98	25.1
	Diploma	79	20.95
	Degree and above	43	11.4
Mothers’ occupational status	Housewife	127	30.6
	Student	9	2.17
	Government employee	117	28.3
	Private work	123	29.7
	Others	38	9.17
Fathers’ occupational status	Farmer	86	22
	Merchant	76	19.5
	Government employee	132	33.7
	Private work	97	24.8
Family size	≤ 3	34	7.8
	4–5	285	65.4
	≥ 6	117	26.8
Monthly income	< 1000	52	11.9
	1000–2000	292	67
	> 2000	92	20.1

Out of the 436 children, 37 (8.5%) were being sick in the last semester and only 140 (32.1%) of school-age children were attending preschool. Majority of study participants 403 (92.44) were traveled to school with in 2.18 km (Additional file 1: Table S1).

**Fig. 1:**
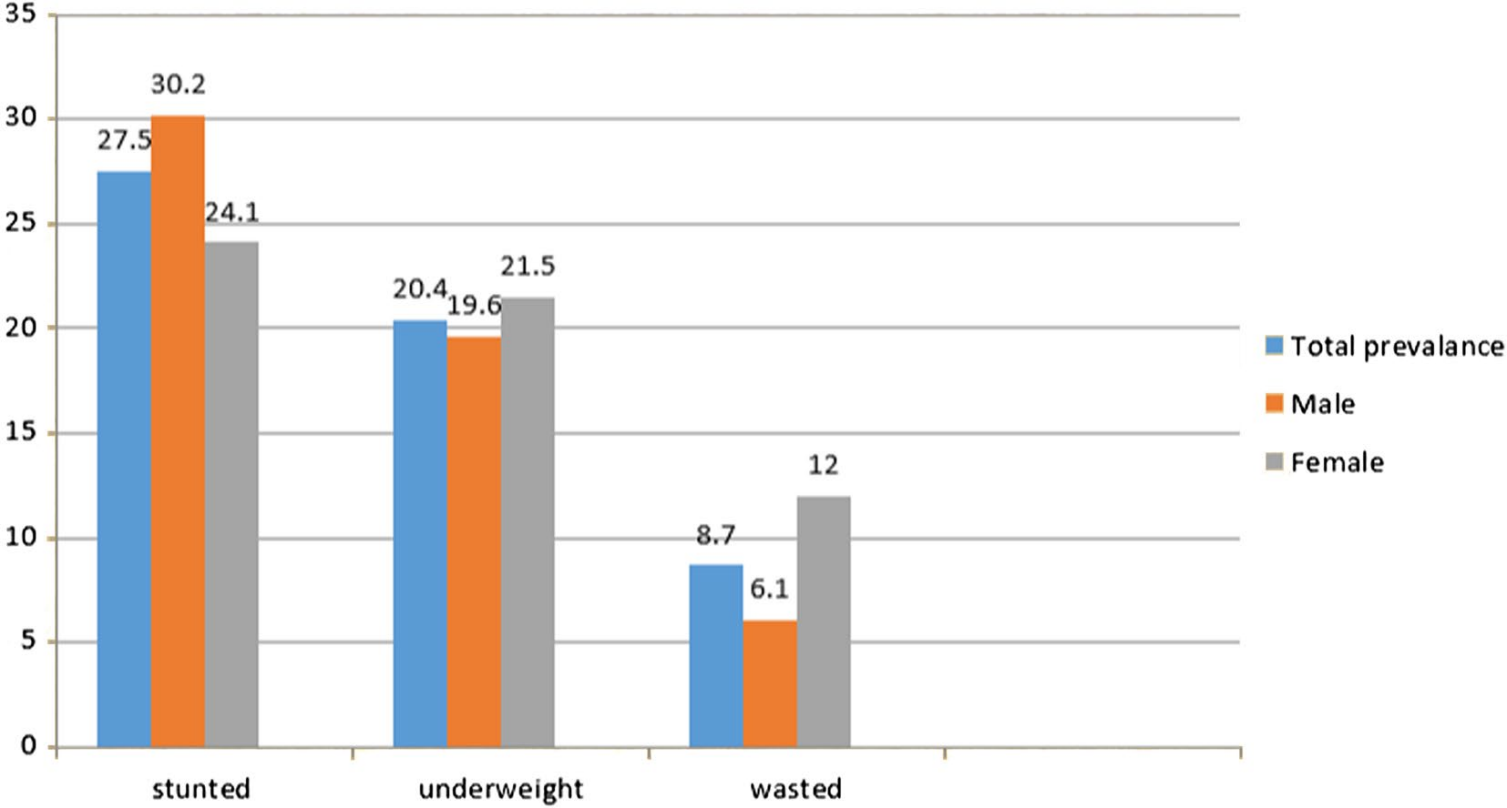
Prevalence of under nutrition by gender among children in Debre Markos town, Northwest, Ethiopia, 2017 (n = 436)

*Level of nutritional status in study participants:* The overall prevalence of stunting, underweight and wasting were 27.5% (95% CI 23.2–31.9%), 20.4% (95% CI 16.5–24.3%) and 8.7% (95% CI 6.2–11.5%) respectively. The percentage of children having any kind of under nutrition (stunting, wasting and underweight) was 56.2%. The prevalence of stunting was significantly higher in males than females (Fig. 1).

*Nutritional status and academic performance:* In comparison of median t-test analysis revealed that there was significant mean difference in all subject average score between children who were having any kind of undernutrition (Additional file 2: Table S2).

After adjusting factors, age, family income, nutritional indicators (WAZ and HAZ) had significant positive associations with academic achievement of students (*p *< 0.05). Age of the child (AOR = 0.177, 95% CI 0.07, 0.4), Monthly income less < 1000.00 birr (AOR = 0.05, 95% CI 0.02, 0.15), stunted children (AOR = 0.21, 95% CI 0.10, 0.43) and under-weight (AOR = 0.63, 95% CI 0.26, 0.84) were significantly associated with academic performance. Students those nutritional status had stunted were 79% less likely to score high academic performance as compared with normal. Students whose nutritional status had under-weight were 37% less likely to score high academic performance compared with their counterparts (Table 2).

**Table 2:** Bi-variable and multivariable logistic regression of variables and academic performance of study participants in Debre Markos town, Northwest, Ethiopia, March, 2017 (n = 436)

Variables	Categories	High (≥ 50%)	Low (< 50%)	COR	AOR	p-value
Age of child (year)	7	20.8	18.8	0.69 (0.39–1.24)	0.437 (0.13–1.47)	0.183
	8	29.4	26.2	0.71 (0.42–1.204)	0.37 (0.14–0.98)	0.047
	9	17.6	34.6	0.32 (0.18–0.55)	0.17 (0.07–0.43)	0.002*
	10	32.2	20.4	1	1	
Grade level	Grade one	33.1	37.7	0.43 (0.21–0.87)	0.66 (0.17–2.48)	0.545
	Grade two	34.3	33	0.51 (0.25–1.1)	0.88 (0.27–2.78)	0.828
	Grade three	18	22	0.41 (0.19–1.86)	0.75 (0.25–2.22)	0.606
	Grade four	14.7	7.3	1	1	
Educational status of mothers	No formal education	18.8	21.5	2.99 (0.74–12.03)	1.26 (0.26–5.9)	0.76
	Primary	40	35.6	3.84 (0.98–5.01)	2.12 (0.46–9.59)	0.32
	Secondary	34.7	28.3	4.19 (1.06–16.6)	2.07 (0.44–9.79)	0.35
	Diploma	5.3	10.5	1.38 (1.38–7.7)	1.45 (0.24–8.64)	0.68
	Degree and above	1.2	4.2	1	1	
Family size	≤ 3	11.8	2.6	1	1	
	4–5	61.6	70.2	0.19 (0.07–0.51)	0.54 (0.08–1.78)	0.17
	≥ 6	26.5	27.2	0.21 (0.07–0.59)	0.21 (0.06–1.6)	0.13
Monthly income	< 1000	4.9	20.9	0.04 (0.02–11)	0.05 (0.02–0.15)	0.001*
	1000–2000	62.9	72.3	0.18 (0.09–0.34)	0.18 (0.08–0.37)	0.001*
	> 2000	32.2	6.8	1	1	
Sickness in last semester	Yes	79.6	64.9	2.107 (1.37–3.23)	0.383 (0.153–1.95)	0.43
	No	20.4	35.1	1	1	
Absenteeism	Non absent	24.9	7.9	3.89 (2.13–7.08)	2.14 (0.96–11.54)	0.08
	Absent	75.1	92.1	1	1	
Study time in home	No	4.5	13.6	0.29 (0.14–0.62)	0.82 (0.45–1.48)	0.51
	Yes	95.5	86.4	1	1	
WAZ	Under weight	10.2	33.5	0.22 (0.13–0.37)	0.63 (0.26–0.84)	0.003*
	Normal	89.8	66.5	1	1	
HAZ	Stunted	16.3	41.9	0.27 (0.17–1.22)	0.21 (0.10–0.43)	0.001*
	Normal	83.7	58.1	1	1	
WHZ	Wasted	4.5	14.1	0.28 (0.13–0.59)	0.80 (0.54–1.2)	0.06
	Normal	95.5	85.9	1	1	

* Significant factors in the multivariable analysis

### Discussions

The aim of this study was to determine the relationship between nutritional status and academic performance among governmental primary school children. In this study, the prevalence of stunting, underweight and wasting were 27.5%, 20.4%, and 8.7% respectively. This finding was comparable with a study done in Zambia reported that 28.9% of stunted, 14.5% of underweight and 3. 9% of wasted [14]. In addition, the prevalence of stunting and wasting in this study was also in line with the findings of Sri Lanka among school-age children which indicated that the prevalence of under-nutrition in the central province was 26.6% stunted and 8.5% wasted [15], and in northwest Ethiopia, 27.1% stunted [16]. In contrast, this finding was higher as compared to other previous studies conducted in Brazil was found (14.9% stunted and 9.7% wasted) [9], in Kenya (24% stunted, 14.9% underweight, 9.7% wasted) [17], in Nicaragua (5% wasted) [18], in eastern Ethiopia (8.9% stunted) [19]. The reason for this observed discrepancy might be due to sociodemographic characteristics, area of sampling and study period.

Regarding factors, the present study revealed that age and monthly income were significant factors for academic performance among primary school children. This finding was consistent with a systematic review and meta-analysis showed that there is a strong association between academic performance and socio-economic status including age [20]. Compromised socio-economic status of a family was statistically associated with poor academic performance in children [21]. Similarly, other studies done in Southeast Ethiopia [22] and in Malaysia [23] reported that minimum wealth indexed score of the family were a positive association with poor academic performance. This might be due to balanced nutritional intake is required for adequate biological functioning affect such complex brain functions as the cognitive processes related academic performance [24].

Moreover, in developing countries macronutrient and micronutrient deficiencies are a devastating problem. Consequently, this obstacle has been either direct or indirect influence on children future of life [25]. Improved nutritional status has been exposed to have a positive and direct impact on academic performance of children [4]. In the current study, under-weight and stunting were associated factors for good academic performance among school-age children. This finding is in line with a study done in Sri Lanka [26] and in Uganda [27] which revealed that child with a high score of WHZ and HAZ had good academic performance as compared to their counterparts. Also, marasmic-kwashiorkor children may acquire delay brain development. Chronic types of malnutrition (stunting) had a negative impact on child cognitive development [20]. Similarly, a study done in Southeast Ethiopia revealed that higher score of HAZ was significantly associated with a higher academic score [20]. In this study, wasting (WHZ) was not statistical association with child academic performance. This non-significance effect might be due to the fact that wasting is acute malnutrition which implies a temporary nutritional disorder that may not negative substantial impact on academic performance [28, 29].

### Conclusion

The study revealed that indicators of undernutrition were prevalent among Debre Markos town primary school children. Age, income, HAZ and WAZ scores showed significant association with academic performance. Therefore, the government should paid attention to implement nutrition screening program and intervention strategy to improve academic performance at primary school children.

## Limitation of the study

Finally, some important limitations of this study was cross-sectional nature of the study could not establish a cause and effect relationship between the dependent and independent variables. The other limitation of the study is that it was done in an urban areas which may inadequate representative for rural area.

## Additional files

**Additional file 1: Table S1.** General characteristics of study participants in Debre Markos town, March, 2017 (n = 436).

**Additional file 2: Table S2.** Prevalence of low educational performance (marks < median of student result of nutritional status of children, Debre Markos, 2017 (n = 436).
