# Supplementary material for: Nutritional status and correlation with academic performance among primary school children, northwest Ethiopia
Source: BMC Res Notes. 2018 Nov 9;11:805. doi: 10.1186/s13104-018-3909-1 (PMC6230243; doi:10.1186/s13104-018-3909-1)
Supplement: Supplementary file 1 — Additional file 1: Table S1. General characteristics of study participants in Debre Markos town, March, 2017 (n = 436). [file 13104_2018_3909_MOESM1_ESM.docx]

**Table S1: General characteristics of study participants in Debre Markos town , March, 2017(n=436).**

| Variable | Category | Frequency  (n:436) | Percent(%) |
| --- | --- | --- | --- |
| Sickness in the first semester | Yes | 37 | 8.5% |
|  | No | 399 | 91.5% |
| Preschool attending | Yes | 140 | 32.1% |
|  | No | 296 | 67.9% |
| Tutorial in home | Yes | 76 | 17.4% |
|  | No | 360 | 82.6% |
| Absent from class | No absent | 260 | 59.6% |
|  | Absent | 176 | 40.4% |
| Distance from home to school | < 2.18 km | 403 | 92.44% |
|  | ≥ 2.18 Km | 33 | 7.56% |
