# Supplementary material for: Nutritional status and correlation with academic performance among primary school children, northwest Ethiopia
Source: BMC Res Notes. 2018 Nov 9;11:805. doi: 10.1186/s13104-018-3909-1 (PMC6230243; doi:10.1186/s13104-018-3909-1)
Supplement: Supplementary file 2 — Additional file 2: Table S2. Prevalence of low educational performance (marks < median of student result of nutritional status of children, Debre Markos, 2017 (n = 436). [file 13104_2018_3909_MOESM2_ESM.docx]

**Table S2: Prevalence of low educational performance (marks < median of student result of nutritional status of children, Debre Markos, 2017(n=436)**

| Characteristics/Variables n | prevalence (%) of academic performance | | p-value |
| --- | --- | --- | --- |
|  | Low | *High* |  |
| Height-for-Age |  |  |  |
| Stunted 120 | 66.7 | 33.3 |  |
| Normal 316 | 35.1 | 64.9 | P<.001 |
| Weight- for- age |  |  |  |
| Underweight 89 | 71.9 | 28.1 |  |
| Normal 347 | 36.6 | 68.4 | P<.001 |
| Weight –for- height |  |  |  |
| Wasted 38 | 71.1 7171.1 | 28.9 | P=0.02 |
| Normal 398 | 41.2 441.241.2 | 58.8 |  |

******Pearson chi- square test TSA. Total subject average*
